# Effect of abdominal binding on respiratory mechanics during exercise in athletes with cervical spinal cord injury

**DOI:** 10.1152/japplphysiol.00218.2014

**Published:** 2014-05-22

**Authors:** Christopher R. West, Victoria L. Goosey-Tolfrey, Ian G. Campbell, Lee M. Romer

**Affiliations:** ^1^Centre for Sports Medicine and Human Performance, Brunel University, United Kingdom; and; ^2^School of Sport, Exercise & Health Sciences, The Peter Harrison Centre for Disability Sport, Loughborough University, United Kingdom

**Keywords:** diaphragm, respiratory muscles, tetraplegia, upper-body exercise, wheelchair exercise

## Abstract

We asked whether elastic binding of the abdomen influences respiratory mechanics during wheelchair propulsion in athletes with cervical spinal cord injury (SCI). Eight Paralympic wheelchair rugby players with motor-complete SCI (C_5_-C_7_) performed submaximal and maximal incremental exercise tests on a treadmill, both with and without abdominal binding. Measurements included pulmonary function, pressure-derived indices of respiratory mechanics, operating lung volumes, tidal flow-volume data, gas exchange, blood lactate, and symptoms. Residual volume and functional residual capacity were reduced with binding (77 ± 18 and 81 ± 11% of unbound, *P* < 0.05), vital capacity was increased (114 ± 9%, *P* < 0.05), whereas total lung capacity was relatively well preserved (99 ± 5%). During exercise, binding introduced a passive increase in transdiaphragmatic pressure, due primarily to an increase in gastric pressure. Active pressures during inspiration were similar across conditions. A sudden, sustained rise in operating lung volumes was evident in the unbound condition, and these volumes were shifted downward with binding. Expiratory flow limitation did not occur in any subject and there was substantial reserve to increase flow and volume in both conditions. V̇o_2_ was elevated with binding during the final stages of exercise (8–12%, *P* < 0.05), whereas blood lactate concentration was reduced (16–19%, *P* < 0.05). V̇o_2_/heart rate slopes were less steep with binding (62 ± 35 vs. 47 ± 24 ml/beat, *P* < 0.05). Ventilation, symptoms, and work rates were similar across conditions. The results suggest that abdominal binding shifts tidal breathing to lower lung volumes without influencing flow limitation, symptoms, or exercise tolerance. Changes in respiratory mechanics with binding may benefit O_2_ transport capacity by an improvement in central circulatory function.

individuals with cervical spinal cord injury (SCI) exhibit restrictive pulmonary dysfunction, characterized by a significant reduction in lung volumes (51, 53). This restrictive defect has been attributed to weakened respiratory muscles (30), reduced compliance of the lung and chest wall (37), and reduced expanding effect of the diaphragm on the lower rib cage owing to increased abdominal wall compliance (44). During exercise, individuals with cervical SCI demonstrate an immediate and sustained rise in end-expiratory and end-inspiratory lung volumes (i.e., dynamic hyperinflation) (41). This rise in operating lung volumes would be expected to increase the elastic work of breathing, impair the capacity of the inspiratory muscles to generate pressure, and reduce the relative contribution of the diaphragm to inspiration (41). Cervical SCI also leads to alterations in cardiovascular function during exercise. With complete cervical SCI, maximal heart rate is usually limited to ∼120–130 beats/min owing to a lack of supraspinal sympathetic drive to the heart (19). Furthermore, vasomotor tone is impaired owing to a lack of descending sympathetic vascular control (26) and low catecholamine spillover (38). Consequently, blood cannot be redistributed effectively during exercise. This has been associated with venous pooling in nonactive vascular beds (43) and may, in turn, restrict O_2_ transport to working muscles by compromising venous return and stroke volume (22). The aforementioned increase in abdominal compliance may further compromise venous return and stroke volume by reducing the abdominothoracic pressure gradient (3, 4).

Previous studies have attempted to increase O_2_ transport in individuals with cervical SCI by using a supine position during arm exercise (18, 21), electrical stimulation of lower-limb muscles (14, 17), and application of lower-body positive pressure by means of an antigravity suit (20, 21, 34). An alternative method has been to apply external compression to the abdomen using an elastic binder. This latter approach has been shown to confer multiple benefits at rest, including increases in vital capacity, expiratory flow, respiratory muscle strength, blood pressure, and stroke volume (47, 52). The effects of abdominal binding on exercise responses have been variable (20, 21). These inconsistencies may have stemmed from differences in exercise protocols, exercise modalities, and subject characteristics. In athletes with cervical SCI, we recently showed that abdominal binding increases the distance covered during a field-based endurance test (50). On the basis of a significant positive correlation between distance covered in the field and peak O_2_ uptake assessed in the laboratory (*r* = 0.75, *P* < 0.05), we considered that the ergogenic effect of binding on endurance performance might have been attributable to an improvement in central circulatory function (50).

The purpose of the present study, therefore, was to better understand the influence of abdominal binding on the acute physiological responses to exercise in athletes with cervical SCI. The specific objective was to determine the effect of abdominal binding on respiratory mechanics during graded wheelchair exercise. Our hypothesis was that abdominal binding would increase intra-abdominal pressure, reduce operating lung volumes, and improve diaphragm function during exercise. We reasoned that these binding-induced changes would improve the circulatory function of the diaphragm, thereby enhancing the overall exercise response through an increase in venous return, cardiac output, and O_2_ transport.

## METHODS

### Subjects

After providing written informed consent, 8 members of the Great Britain wheelchair rugby squad (1 woman) participated in the study. The subjects had traumatic SCI (2 C_5_, 5 C_6_, 1 C_7_) and motor-complete lesions [American Spinal Injury Association Impairment Scale A (*n* = 7) or B (*n* = 1)]. Subject characteristics (mean ± SD) were: age 29 ± 2 yr, stature 1.79 ± 0.10 m, body mass 67 ± 15 kg, and time postinjury 9 ± 3 yr. None of the subjects smoked, had a history of cardiopulmonary disease, or were taking medications known to influence the exercise response. At the time of study the subjects were performing at least 15 h/wk of endurance, resistance, and sport-specific training. All of the subjects had taken part in our previous binding studies (50, 52) and were familiar with treadmill exercise testing. The primary outcome measures in the current study do not overlap with previous analyses. Subjects were required to refrain from strenuous exercise for 48 h before testing. Caffeine and alcohol were prohibited for 12 and 24 h, respectively, and no food was allowed within 2 h before testing. Upon arrival at the laboratory, the subjects emptied their bladders to reduce the likelihood of autonomic dysreflexia (12).

### Study Design

**Fig. 1.**
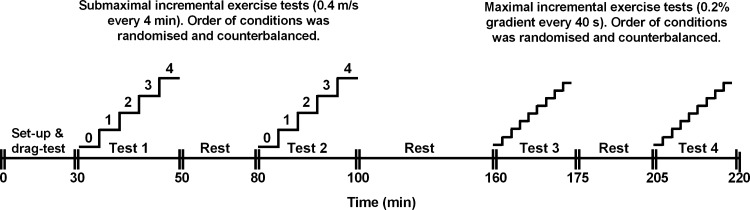
Experimental overview for submaximal and maximal exercise tests.

Subjects visited the laboratory on two separate occasions over a period of ∼1 wk. Visit 1 included an evaluation of pulmonary function and static respiratory pressures. Visit 2 included submaximal and maximal exercise tests on a treadmill (Fig. 1). The assignment of conditions (unbound and bound) was randomized and counterbalanced. The order of exercise tests was sequential (i.e., submaximal exercise in both conditions, maximal exercise in both conditions). The subjects rested for 30 min between conditions and 60 min between tests. The conditions could not be blinded, but the participants were unaware of the experimental hypotheses and expected outcomes of the study. Cardiopulmonary, metabolic, and perceptual responses were assessed during the submaximal and maximal exercise tests. Because of the invasiveness of the procedures (balloon catheters) and the duration of the experimental visit (∼4 h) it was neither feasible nor ethical to measure intrathoracic pressures in both tests; therefore, respiratory mechanics and ventilatory constraint were assessed during submaximal exercise only. The subjects performed all tests in their own sports wheelchair. Gloves were worn for the exercise tests and leg/chest straps if needed. The study procedures received institutional ethical approval and conformed to the Declaration of Helsinki.

### Procedures

#### Abdominal binding.

The binder (493R Universal Back Support; McDavid, Woodridge, IL) incorporated a semirigid neoprene back panel with six plastic stays (100% neoprene rubber), flexible side-panels (90% nylon, 10% Lycra), and a flexible neoprene front panel with double Velcro fastening. The binder was individually sized and fitted in the upright position with the upper edge just beneath the costal margin so that the binder interfered minimally with rib cage movement. An inflatable rubber reservoir with a known volume of air was connected to a digital manometer (C9553; JMW, Harlow, UK) and placed between the binder and the anterior abdominal wall. The binder tightness was adjusted until end-expiratory gastric pressure was approximately twice that in the unbound condition; this level of binding has been shown to optimize resting cardiopulmonary function (52) and improve field-based endurance performance (50). The corresponding abdominal-wall pressure was used to set the binder tightness for the maximal exercise test.

#### Pulmonary function and static respiratory pressures.

Pulmonary volumes, capacities, and flows were assessed using spirometry and body plethysmography (Zan 530; nSpire Health, Oberthulba, Würzburg, Germany) (24, 32, 48). Maximum static inspiratory and expiratory pressures were measured at the mouth (MicroRPM; CareFusion, Basingstoke, UK) from functional residual capacity and total lung capacity, respectively (15).

#### Exercise tests.

Exercise tests were performed on a motorized treadmill with a moving rail to prevent falls (Saturn 300/125r; HP Cosmos, Nussdorf-Traunstein, Germany). The submaximal test consisted of a steady-state resting period followed by four stages, starting at 1.6 m/s and incrementing by 0.4 m/s every 4 min with a 30-s break between stages (27). The maximal test consisted of a fixed speed, chosen according to the responses elicited during the submaximal test, and an increase in gradient of 0.2% every 40 s. The maximal test was terminated when subjects were unable to maintain the treadmill speed, i.e., when they touched the spring of the safety rail for a third time. Standardized verbal encouragement was given throughout the tests, but no information was provided regarding speed, time, or physiological response. Push rate was freely chosen and assessed based on the number of hand-to-rim contacts recorded during the final minute of each stage. After the maximal test the subjects rested for 2 min and then performed an active recovery at low exercise intensity for 5 min. Pretest values were not different at baseline, indicating that the time between tests ensured a full recovery. Power output for each subject-wheelchair combination was determined prior to exercise using a separate drag-test (45).

#### Cardiopulmonary, metabolic, and perceptual responses.

Ventilatory and pulmonary gas exchange variables were assessed breath-by-breath using an online system (Oxycon Pro; Jaeger, Höchberg, Germany). Arterial O_2_ saturation was estimated using a pulse oximeter with earlobe sensor (PalmSAT 2500; Nonin Medical, Minneapolis, MN). Heart rate was assessed beat-by-beat via telemetry (Vantage NV; Polar Electro Oy, Kempele, Finland). Earlobe capillary blood was sampled immediately before each test and after each submaximal stage for the determination of lactate concentration in hemolyzed whole-blood (1500 SPORT; YSI Inc, Yellow Springs, OH). After the maximal test, blood was sampled at 0.5, 2, 4, 6, and 8 min and peak lactate concentration was defined as the highest value. Ratings of dyspnea (respiratory discomfort) and arm discomfort were obtained immediately after each stage using Borg's modified 0–10 category-ratio scale (8).

#### Respiratory mechanics and ventilatory constraint.

Gastric pressure (P_ga_) and esophageal pressure (P_es_) were measured continuously using previously described procedures (41). Transdiaphragmatic pressure (P_di_) was obtained by electronic subtraction of P_es_ from P_ga_. An analog airflow signal from the online gas analysis system was simultaneously input into the data acquisition system and aligned to the pressure signals based on the sampling delay for flow. Maximal static inspiratory efforts from functional residual capacity were performed at resting baseline to obtain maximum values for P_di_, P_ga_, and P_es_. To evaluate the *passive* increase in pressures introduced by application of the binder we report end-expiratory and end-inspiratory values for P_di_, P_ga_, and P_es_. To permit comparison of the *active* pressures generated in both conditions we report inspiratory pressure swings from end-expiratory values, calculated as peak-to-peak (P_di,tidal_, P_ga,tidal_, P_es,tidal_) and integrated pressure-time product (PTP_di_, PTP_ga_, PTP_es_). Dynamic lung compliance during inspiration was calculated as the ratio of tidal volume to P_es,tidal_ (36). To determine the likelihood of inspiratory muscle fatigue, the tension-time index of the diaphragm (TTI_di_) was calculated as P̄_di_/P_di,max_·T_I_/T_TOT_, where P̄_di_ is mean transdiaphragmatic pressure integrated over inspiration with reference to the end-expiratory level, P_di,max_ is maximum transdiaphragmatic pressure, T_I_ is inspiratory time, and T_TOT_ is total breath time (7).

The degree of ventilatory constraint was assessed by measuring changes in operating lung volumes, expiratory flow limitation, inspiratory flow reserve, and the ratio of minute ventilation (V̇_E_) to the maximal estimated ventilation for a given breathing pattern (V̇_ECAP_), as described previously (5, 23). Briefly, changes in operating lung volumes [end-expiratory lung volume (EELV) and end-inspiratory lung volume (EILV)] were assessed by measuring inspiratory capacity (IC) relative to total lung capacity (TLC), immediately before exercise and during the final 30 s of each submaximal exercise stage [EELV = TLC − IC; EILV = (TLC − IC) + tidal volume]. Peak inspiratory P_es_ during the IC maneuver was not significantly different across exercise stages in either condition, indicating good reproducibility of maximal efforts for assessment of operating lung volumes. The degree of expiratory flow limitation, if present, was defined as the percent of the tidal flow-volume loop that met or exceeded the expiratory portion of the largest maximal flow-volume loop obtained before or <2 min after exercise (highest sum of FEV_1_ and FVC). Inspiratory flow reserve (IFR) was expressed as the peak inspiratory flow generated during tidal breathing relative to that achieved during the maximal flow-volume maneuver at the same lung volume. The level of ventilation relative to a theoretical maximal ventilatory capacity (*V̇*_e_/V̇_e__CAP_) was also determined, where V̇_ECAP_ represents the total area under the expiratory flow curve between EILV and EELV.

### Data Analysis

Cardiopulmonary data at rest and during submaximal exercise were averaged over 30-s epochs. To avoid breath contamination from paired IC measurements, the first 30 s of every 4th min of submaximal exercise was analyzed. The 30 s of data used for analysis was filtered to remove outlying breaths, defined as any breath deviating by more than three standard deviations from the mean T_TOT_ during the preceding 5 breaths. Peak cardiopulmonary responses are reported as the highest 30-s average. To determine the degree of expiratory flow limitation, an average breath was constructed for the selected 30-s period by splitting each breath into equal time segments. The number of time segments was based on the mean T_TOT_ with a resolution of 0.01 s. A flow-volume loop was then constructed from the average breath and placed at EELV inside the maximal flow-volume loop for the subsequent assessment of ventilatory constraint.

### Statistics

Analyses were performed using SPSS 16.0 for Windows (IBM, Chicago, IL). Data were checked for normality using the Kolmogorov-Smirnov test and homogeneity of variance using Levene's statistic. None of the assumptions underlying parametric testing was violated. Submaximal exercise data were assessed for differences using two-factor (condition × time) repeated-measures ANOVA. Where a significant interaction effect was detected, post hoc analysis was carried out using Bonferroni-corrected pairwise comparisons. Pulmonary function and maximal exercise data were assessed for differences using two-tailed paired *t*-tests. Pearson's correlation coefficient was calculated to establish correlations between heart rate (dependent variable) and O_2_ uptake by subject. The slope and intercept of the equations describing each of these correlations were assessed using linear regression analysis. Critical significance level α was set at 0.05. Values are presented as means ± SD unless stated otherwise.

## RESULTS

### Pulmonary Function and Static Respiratory Pressures

Pulmonary function and static respiratory pressures are summarized in Table 1. Abdominal binding increased vital capacity, whereas decreases were noted for functional residual capacity and residual volume. Total lung capacity was relatively well preserved. Forced expiratory volume in 1 s was increased with binding. Maximum inspiratory mouth pressure was not affected by binding, whereas maximum expiratory mouth pressure was increased.

**Table 1.** Effect of abdominal binding on pulmonary function and static respiratory pressures

	Unbound	Bound	%Δ
TLC, liters	5.40 ± 1.15	5.38 ± 1.29	−1 ± 5
	(77 ± 9)	(76 ± 10)	
FRC, liters	3.25 ± 0.92	2.68 ± 1.01*	−19 ± 11
	(98 ± 23)	(81 ± 27)	
RV, liters	1.83 ± 1.01	1.42 ± 0.99*	−23 ± 32
	(109 ± 59)	(83 ± 57)	
IC, liters	2.42 ± 0.61	2.91 ± 0.69*	21 ± 7
	(65 ± 7)	(78 ± 9)	
IRV, liters	1.70 ± 0.53	2.20 ± 0.58*	32 ± 14
ERV, liters	1.08 ± 0.38	1.03 ± 0.30	−2 ± 16
	(67 ± 21)	(64 ± 16)	
VC, liters	3.49 ± 0.97	3.93 ± 0.94*	14 ± 9
	(65 ± 10)	(74 ± 10)	
FEV_1_, liters	2.96 ± 0.81	3.33 ± 0.72*	15 ± 14
	(68 ± 12)	(77 ± 9)	
FEV_1_/VC, %	84.0 ± 9.8	86.4 ± 7.6	3 ± 7
	(102 ± 11)	(105 ± 9)	
PEF, l/s	5.8 ± 1.5	6.2 ± 1.6	7 ± 13
	(60 ± 11)	(64 ± 12)	
MEF_25–75_, l/s	3.18 ± 1.05	3.81 ± 1.00	28 ± 40
	(65 ± 21)	(78 ± 19)	
MVV_12_, l/min	109 ± 29	111 ± 28	3 ± 13
	(68 ± 17)	(69 ± 18)	
P_Imax_, cmH_2_O	−98 ± 45	−103 ± 43	9 ± 20
	(86 ± 33)	(91 ± 32)	
P_Emax_, cmH_2_O	59 ± 18	73 ± 21*	26 ± 34
	(43 ± 9)	(53 ± 12)	

Values are means ± SD for 8 subjects.

TLC, total lung capacity; FRC, functional residual capacity; RV, residual volume; IC, inspiratory capacity; IRV, inspiratory reserve volume; ERV, expiratory reserve volume; VC, vital capacity; FEV_1_, forced expiratory volume in 1 s; PEF, peak expiratory flow; MEF_25–75_, midexpiratory flow between 25 and 75% of VC; MVV_12_, maximal voluntary ventilation in 12 s; P_Imax_, maximum static inspiratory pressure from FRC; P_Emax_, maximum static expiratory pressure from TLC. Values in parentheses are percent of able-bodied predicted values for pulmonary volumes, capacities, and flows (35); MVV (16); and respiratory pressures (10). Predicted values for ERV and IC were derived from differences between corresponding predicted values for FRC and RV, and between TLC and FRC, respectively (35).

* *P* < 0.05.

### Cardiopulmonary, Metabolic, and Perceptual Responses

**Fig. 2.**
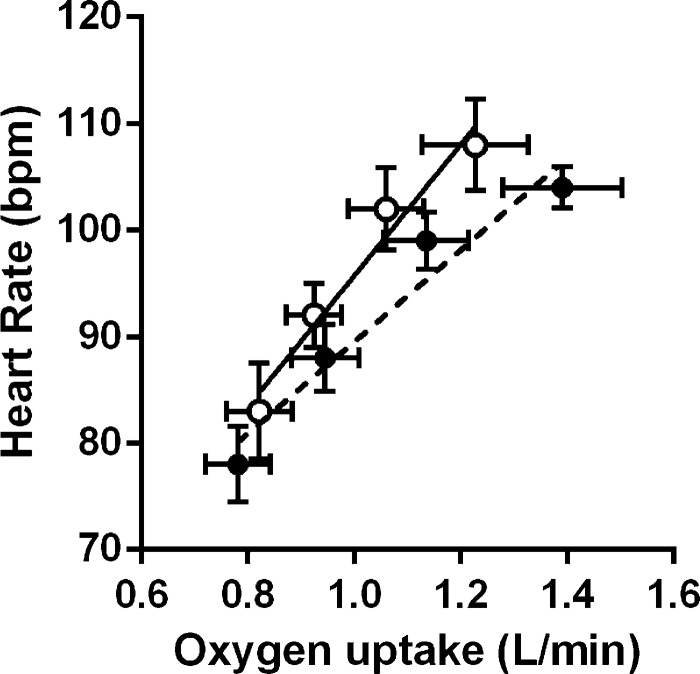
O_2_ uptake/heart rate slopes in the bound (dashed line) and unbound condition (solid line) for measurements during each stage of the submaximal exercise test. Slopes were less steep in the bound condition (*P* < 0.05); see text for details. Data are means ± SE for 8 subjects.

Responses during the submaximal exercise test are summarized in Table 2. In the unbound condition, the test elicited a wide range of values relative to peak: V̇o_2_ (64–95%), V̇_E_ (46–83%), and heart rate (69–90%). There were no differences in ventilation or breathing pattern across conditions. The timing (T_I_/T_TOT_) and drive (V_T_/T_I_) components of ventilation were also not different across conditions. There was a significant interaction effect between condition and time for V̇o_2_ (*P* = 0.002) and blood lactate concentration (*P* = 0.010), whereby V̇o_2_ was elevated (8%) and lactate was reduced (19%) in the bound condition during the final stage of the test. The O_2_ pulse (V̇o_2_/heart rate) was also elevated in the bound condition during the final stage (13.4 ± 2.3 vs. 12.3 ± 2.3 ml/beat, *P* = 0.04). The V̇o_2_/heart rate relationship for measurements during submaximal exercise are shown in Fig. 2. The relationships were linear, with high correlations in the unbound and bound condition (*r* = 0.933 ± 0.069 and 0.967 ± 0.032, respectively; both *P* < 0.05). The slopes were less steep in the bound condition (47 ± 24 vs. 62 ± 35 ml/beat, *P* = 0.022), whereas the intercepts were not different (42 ± 20 vs. 34 ± 22 beat/min, *P* = 0.149). Perceptual intensities were similar across conditions.

**Table 2.** Effect of abdominal binding on cardiopulmonary, metabolic, and perceptual responses at rest and during submaximal incremental wheelchair propulsion

Effect	Baseline	Stage 1	Stage 2	Stage 3	Stage 4
Power output, W					
UB	0	20.2 ± 4.5	25.1 ± 5.6	30.1 ± 6.7	35.9 ± 7.9
B	0	20.2 ± 4.5	25.1 ± 5.6	30.1 ± 6.7	35.9 ± 7.9
Push rate, /min					
UB	0	51 ± 11	53 ± 11	63 ± 16	61 ± 14
B	0	49 ± 10	53 ± 11	61 ± 14	60 ± 14
V̇o_2_, l/min‡					
UB	0.32 ± 0.07	0.82 ± 0.17	0.92 ± 0.15	1.07 ± 0.21	1.22 ± 0.26
B	0.27 ± 0.07	0.78 ± 0.17	0.95 ± 0.18	1.13 ± 0.22	1.39 ± 0.26*
V̇co_2_, l/min					
UB	0.27 ± 0.06	0.72 ± 0.16	0.85 ± 0.16	1.01 ± 0.18	1.29 ± 0.23
B	0.25 ± 0.08	0.68 ± 0.17	0.85 ± 0.15	1.06 ± 0.22	1.29 ± 0.27
V̇_E_, l/min					
UB	9.3 ± 2.3	21.2 ± 4.5	25.6 ± 4.9	30.0 ± 5.9	38.4 ± 7.7
B	9.5 ± 3.5	20.8 ± 4.4	26.0 ± 4.7	32.2 ± 7.4	37.3 ± 10.3
*f*_R_, breaths/min					
UB	15.5 ± 3.3	28.2 ± 5.6	34.9 ± 7.0	37.4 ± 9.1	38.6 ± 8.2
B	14.0 ± 2.8	31.0 ± 6.9	35.9 ± 6.6	40.7 ± 7.2	40.2 ± 10.0
V_T_, liters					
UB	0.61 ± 0.16	0.87 ± 0.19	0.85 ± 0.17	0.93 ± 0.18	1.01 ± 0.17
B	0.72 ± 0.32	0.84 ± 0.31	0.84 ± 0.24	0.90 ± 0.22	0.99 ± 0.21
T_I_/T_TOT_					
UB	0.45 ± 0.03	0.48 ± 0.05	0.45 ± 0.04	0.46 ± 0.05	0.48 ± 0.02
B	0.44 ± 0.06	0.45 ± 0.04	0.47 ± 0.04	0.51 ± 0.06	0.47 ± 0.03
V_T_/T_i_, l/s					
UB	0.31 ± 0.09	0.66 ± 0.15	0.88 ± 0.20	0.99 ± 0.30	1.23 ± 0.23
B	0.30 ± 0.09	0.73 ± 0.16	0.86 ± 0.18	0.94 ± 0.53	1.23 ± 0.32
SpO_2_, %					
UB	97 ± 1	97 ± 2	98 ± 2	97 ± 3	96 ± 3
B	97 ± 1	97 ± 2	97 ± 2	96 ± 3	97 ± 3
Heart rate, beats/min					
UB	60 ± 9	83 ± 11	92 ± 9	102 ± 10	108 ± 10
B	58 ± 11	78 ± 11	88 ± 9	99 ± 8	104 ± 6
[La^−^]_B_, mmol/l‡					
UB	0.7 ± 0.2	0.6 ± 0.2	0.8 ± 0.3	1.4 ± 0.6	2.1 ± 1.2
B	0.7 ± 0.2	0.6 ± 0.1	0.6 ± 0.2	1.0 ± 0.3	1.5 ± 0.8*
RPE (dyspnea)					
UB	0	1.1 ± 0.9	2.2 ± 0.8	3.3 ± 1.4	3.7 ± 0.8
B	0	1.3 ± 0.9	2.3 ± 0.9	3.3 ± 1.3	3.4 ± 1.3
RPE (arm discomfort)					
UB	0	1.4 ± 0.9	2.3 ± 0.6	4.1 ± 1.1	4.9 ± 1.9
B	0	1.4 ± 0.8	2.3 ± 0.6	3.7 ± 0.7	4.4 ± 1.0

Values are means ± SD for 8 subjects.

UB, unbound; B, bound; V̇o_2_, O_2_ uptake; V̇co_2_, CO_2_ output; V̇_E_, minute ventilation; *f*_R_, respiratory frequency; V_T_, tidal volume; T_i_/T_tot_, inspiratory duty cycle; V_T_/T_i_, mean inspiratory flow; SpO_2_, arterial O_2_ saturation; [La^−^]_B_, blood lactate concentration; RPE, ratings of perceived exertion.

‡ Significant interaction effect (*P* < 0.05).

* Significant post hoc pairwise comparison (*P* < 0.05).

Responses during the maximal exercise test are summarized in Table 3. Peak power output and push rate were not different across conditions. Peak V̇o_2_ was increased by 12% with binding (*P* = 0.001), yet peak values for heart rate and minute ventilation were similar across conditions. Thus, peak O_2_ pulse was also significantly elevated in the bound condition, whereas, in general, ventilatory equivalents for O_2_ (and CO_2_) were lower. Peak blood lactate concentration was reduced by 16% in the bound condition (*P* = 0.052). Perceptual intensities were similar across conditions.

**Table 3.** Effect of abdominal binding on peak cardiopulmonary, metabolic, and perceptual responses

	Unbound	Bound	*P* value
Power output, W	49 ± 12	50 ± 13	0.980
Push rate, /min	61 ± 13	60 ± 13	0.918
V̇O_2_, l/min	1.29 ± 0.33	1.43 ± 0.35	0.001*
V̇O_2_, ml/kg/min	19.0 ± 2.1	21.2 ± 2.8	0.001*
V̇co_2_, l/min	1.38 ± 0.36	1.54 ± 0.35	0.155
RER	1.08 ± 0.12	1.08 ± 0.13	0.985
V̇_E_, l/min	48.9 ± 14.1	46.1 ± 8.7	0.528
*f*_R_, breaths/min	54 ± 14	53 ± 15	0.838
V_T_, l	0.94 ± 0.21	0.92 ± 0.24	0.709
T_i_/T_tot_	0.48 ± 0.04	0.52 ± 0.06	0.074
V_T_/T_i_, l/s	1.70 ± 0.67	1.70 ± 0.70	0.978
V̇_E_/V̇o_2_	39.0 ± 10.2	33.0 ± 6.0	0.067
V̇_E_/V̇co_2_	35.6 ± 6.1	30.6 ± 4.4	0.012*
P_ET_CO_2_, mmHg	35.5 ± 5.8	37.5 ± 8.0	0.232
SpO_2_, %	95 ± 3	95 ± 3	0.949
Heart rate, beats/min	120 ± 12	122 ± 13	0.534
V̇o_2_/heart rate, ml/beat	10.7 ± 3.1	12.4 ± 3.2	0.001*
[La^−^]_B_, mmol/l	4.6 ± 1.2	3.8 ± 1.0	0.052
RPE (dyspnea)	7.0 ± 2.7	7.1 ± 2.9	0.917
RPE (arm discomfort)	7.5 ± 2.0	7.4 ± 2.0	0.919

Values are means ± SD for 8 subjects.

* Significant difference between conditions (*P* < 0.05).

### Respiratory Mechanics and Ventilatory Constraint

**Fig. 3.**
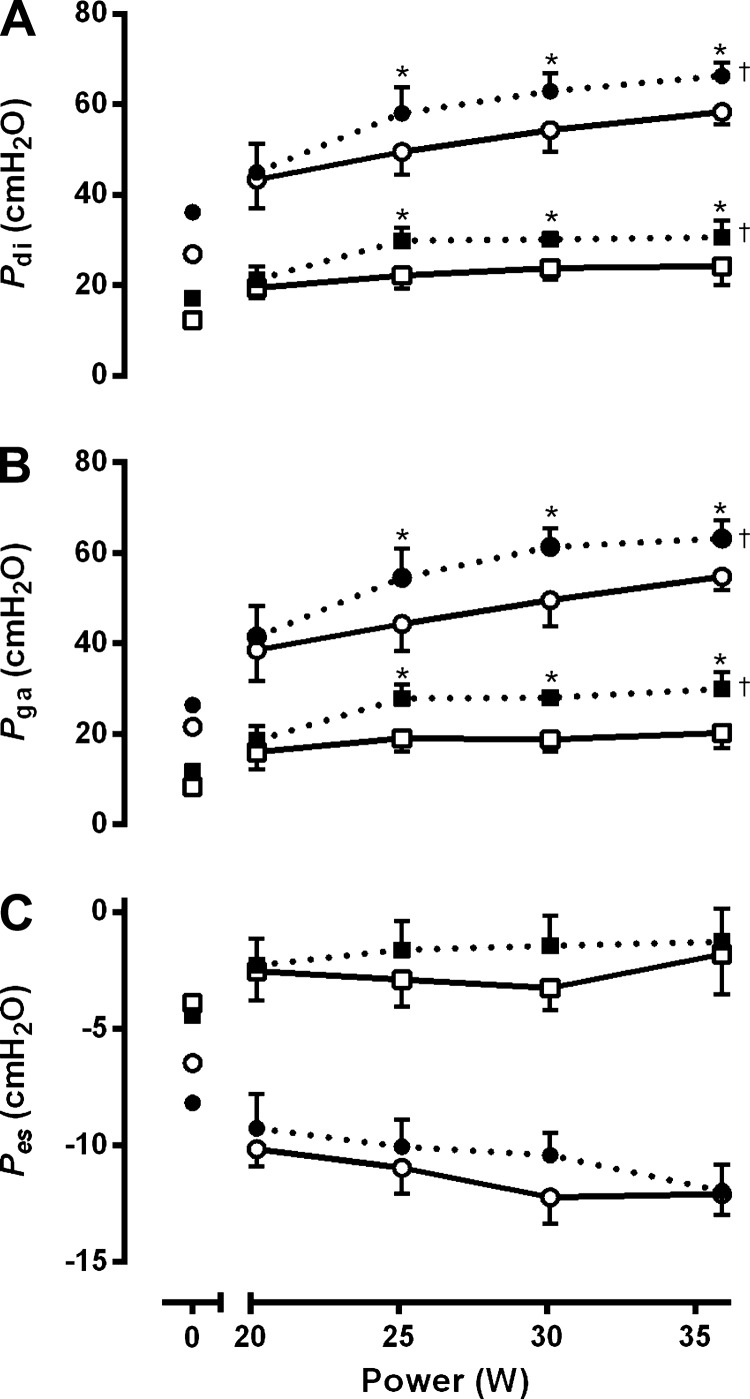
End-expiratory (squares) and end-inspiratory (circles) transdiaphragmatic pressure (*A*), gastric pressure (*B*), and esophageal pressure (*C*) at rest and during submaximal wheelchair propulsion in the bound (dashed lines) and unbound condition (solid lines). Note that end-expiratory and end-inspiratory transdiaphragmatic and gastric pressures were elevated throughout exercise in the bound condition. Data are means ± SE for 7 subjects. †Significant main effect for condition (*P* < 0.05). *Significant post hoc pairwise comparison (*P* < 0.05).

Pressure-derived measurements of respiratory mechanics and ventilatory constraint are reported for seven subjects, because one subject could not tolerate the balloon catheters. End-expiratory and end-inspiratory pressures during the submaximal exercise test are shown in Fig. 3. In the unbound condition, end-expiratory and end-inspiratory P_di_ increased sharply from baseline to the first stage of exercise. End-inspiratory P_di_ continued to increase throughout exercise, whereas end-expiratory P_di_ increased initially and leveled-off thereafter. Both pressures were significantly elevated with application of the binder, primarily because of an increase in the P_ga_ contribution.

Additional indices of respiratory mechanics and ventilatory constraint are summarized in Table 4. Dynamic inspiratory pressures (peak-to-peak and integrated) increased progressively throughout exercise, but were not different across conditions. Dynamic lung compliance fell from baseline to the first stage of exercise then remained stable through to the final stage. Dynamic lung compliance was slightly higher in the bound condition during the latter stages of exercise but did not reach statistical significance. In the unbound condition, TTI_di_ increased progressively throughout exercise due almost entirely to the aforementioned increase in tidal transdiaphragmatic pressure. There was no effect of binding on breath timing, but a slight increase in the maximum pressure-generating capacity of the diaphragm (unbound 125 ± 49 vs. bound 138 ± 32 cmH_2_O, *P* = 0.207) resulted in a trend toward a binding-induced reduction in TTI_di_ (0.20 vs. 0.16 for final stage).

**Table 4.** Effect of abdominal binding on respiratory mechanics and ventilatory constraint at rest and during submaximal incremental wheelchair propulsion

Effect	Baseline	Stage 1	Stage 2	Stage 3	Stage 4
P_di,tidal_, cmH_2_O					
UB	16.0 ± 6.9	29.9 ± 12.8	33.5 ± 11.5	40.9 ± 12.8	44.4 ± 10.1
B	20.5 ± 6.0	35.3 ± 16.7	39.0 ± 17.3	39.7 ± 14.2	43.9 ± 14.4
P_ga,tidal_, cmH_2_O					
UB	13.3 ± 6.6	22.7 ± 10.8	25.4 ± 10.0	31.9 ± 10.6	35.5 ± 7.6
B	16.7 ± 5.6	27.5 ± 14.5	29.5 ± 13.7	31.5 ± 11.5	35.4 ± 11.8
P_es,tidal_, cmH_2_O*					
UB	−2.7 ± 0.7	−7.2 ± 2.5	−8.1 ± 2.6	−8.9 ± 3.0	−10.4 ± 4.3
B	−3.8 ± 1.8	−7.9 ± 3.1	−9.5 ± 5.4	−8.9 ± 4.0	−9.9 ± 5.3
PTP_di_, cmH_2_O·s/min					
UB	225 ± 123	347 ± 148	419 ± 137	467 ± 224	461 ± 231
B	287 ± 137	420 ± 244	470 ± 242	515 ± 136	514 ± 204
PTP_ga_, cmH_2_O·s/min					
UB	187 ± 123	249 ± 132	304 ± 132	328 ± 183	304 ± 211
B	232 ± 131	314 ± 189	316 ± 165	375 ± 109	454 ± 127
PTP_es_, cmH_2_O·s/min					
UB	−38 ± 12	−98 ± 53	−115 ± 48	−135 ± 65	−157 ± 44
B	−55 ± 24	−106 ± 79	−135 ± 65	−140 ± 72	−161 ± 86
C_L,dyn_, ml/cmH_2_O					
UB	184 ± 47	118 ± 58	98 ± 46	103 ± 51	98 ± 54
B	162 ± 45	113 ± 57	114 ± 51	117 ± 79	123 ± 79
TTI_di_					
UB	0.070 ± 0.029	0.109 ± 0.044	0.137 ± 0.063	0.147 ± 0.059	0.203 ± 0.115
B	0.071 ± 0.029	0.074 ± 0.037	0.111 ± 0.043	0.128 ± 0.055	0.159 ± 0.085
IRV/TLC, %					
UB	39 ± 9	27 ± 3	28 ± 5	21 ± 5	17 ± 10
B	43 ± 13	33 ± 9	31 ± 7	29 ± 6	26 ± 4
IFR, % capacity					
UB	6 ± 2	20 ± 13	27 ± 15	26 ± 11	34 ± 18
B	10 ± 2	20 ± 7	28 ± 15	29 ± 11	34 ± 14
V̇_E_/V̇_ECAP_, %					
UB	10 ± 6	17 ± 5	26 ± 9	24 ± 7	40 ± 26
B	15 ± 10	18 ± 9	28 ± 16	23 ± 8	31 ± 16

Values are means ± SD for 7 subjects.

UB, unbound; B, bound; P_di,tidal_, inspiratory tidal transdiaphragmatic pressure; P_ga,tidal_, inspiratory tidal gastric pressure; P_es,tidal_, inspiratory tidal esophageal pressure; PTP_di_, diaphragm pressure-time product; PTP_ga_, gastric pressure-time product; PTP_es_, esophageal pressure-time product; C_L,dyn_, dynamic lung compliance; TTI_di_, inspiratory diaphragm tension-time index; IRV/TLC, index of change in end-inspiratory lung volume; IFR, inspiratory flow reserve; V̇_E_/V̇_ECAP_, ventilatory capacity calculated from a theoretical maximal exercise ventilation based on the maximal available expiratory airflow over the range of the tidal breath placed at the measured end-expiratory lung volume.

* Significant main effect for condition (*P* < 0.05).

**Fig. 4.**
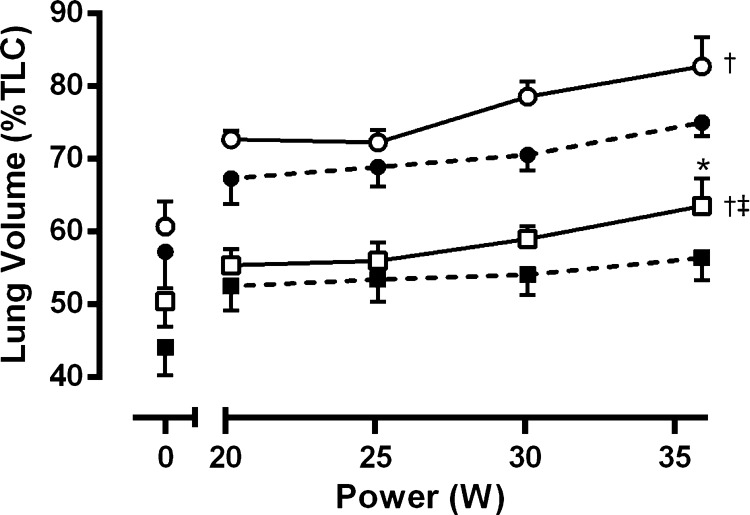
End-expiratory (squares) and end-inspiratory (circles) lung volume at rest and in response to submaximal wheelchair propulsion in the bound (dashed lines, closed symbols) and unbound condition (solid lines, open symbols). Note the immediate and progressive increase from resting values in operating lung volumes (i.e., dynamic hyperinflation) and the downward shift in lung volumes in response to abdominal binding. Data are means ± SE for 7 subjects. †Significant main effect for condition (*P* < 0.05). ‡Significant interaction effect (*P* < 0.05). *Significant post hoc pairwise comparison (*P* < 0.05).

**Fig. 5.**
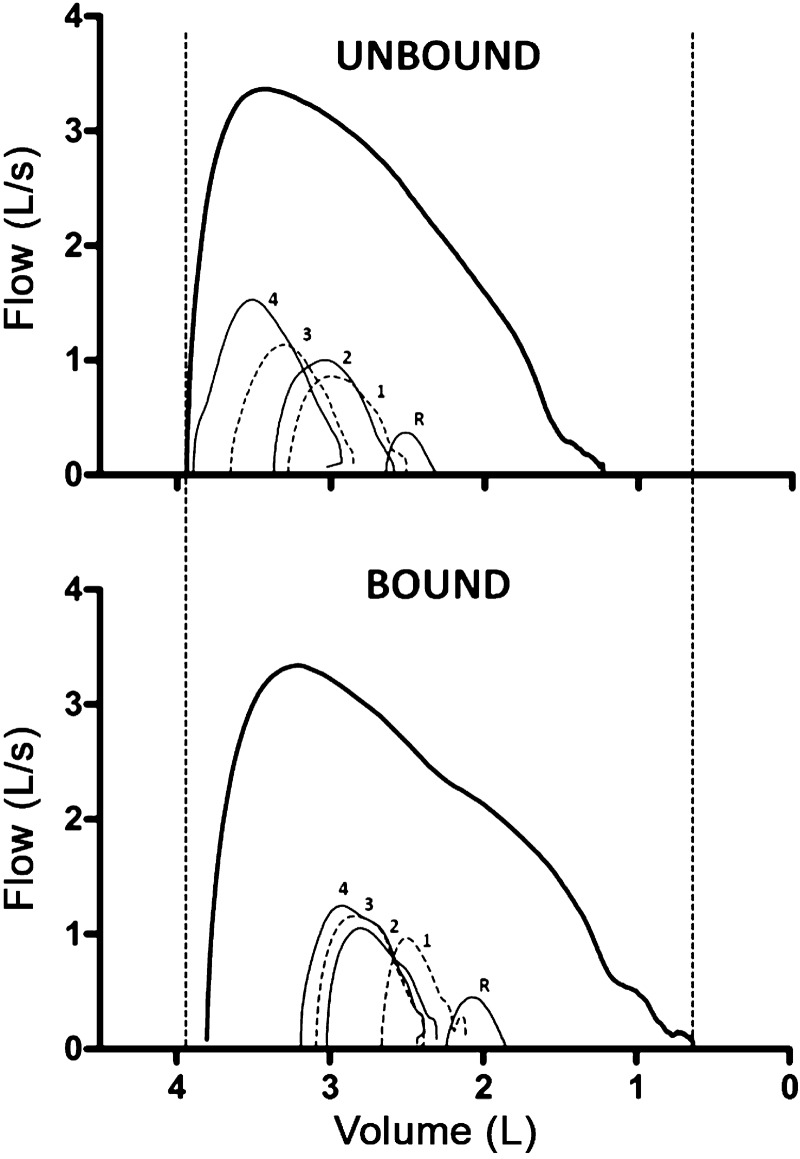
Maximal and tidal flow-volume curves at rest and during the submaximal exercise test for a single subject in the unbound and bound condition. Each tidal flow-volume curve is ensemble-averaged over 30 s of resting baseline (R) and over the first 30 s of the final minute of each exercise stage (1–4). Note the leftward shift of the tidal flow-volume curves as exercise progresses, the rightward shift of the tidal flow-volume curves and concomitant increases in inspiratory reserve volume with binding, and the increase in vital capacity and maximal midexpiratory flows. Vertical dotted lines indicate the binding-induced changes in total lung capacity (*left*) and residual volume (*right*).

Operating lung volumes at rest and during exercise are shown in Fig. 4. In the unbound condition, there was a sharp rise in EELV and EILV from rest to the first stage of exercise and a more gradual increase through to the final stage. Both volumes were shifted to a lower percentage of total lung capacity in the bound condition (−7 ± 2% for EELV, *P* = 0.017; −8 ± 2% for EILV, *P* = 0.035), and the rates of rise were reduced. During the final stage in the unbound condition, EILV averaged 83% of total lung capacity with three subjects exceeding 90%. With binding, EILV was reduced to less than 80% of total lung capacity in all subjects. There was no encroachment of the tidal flow-volume curves on the maximum flow-volume envelope in any subject (e.g., Fig. 5). Furthermore, there was substantial reserve for increasing flow and volume as indicated by the low values for IFR and V̇_E_/V̇_ECAP_, respectively (Table 4).

## DISCUSSION

This study investigated the influence of abdominal binding on respiratory mechanics during wheelchair exercise in highly trained athletes with cervical SCI. The main finding was that binding induced passive increases in intra-abdominal pressure that resulted in a shift of tidal breathing to lower lung volumes with no effect on expiratory flow limitation, symptoms, or exercise tolerance. The binding-induced changes in intra-abdominal pressure were accompanied by increases in whole body O_2_ uptake and decreases in systemic blood lactate at high relative intensities of exercise (≥95% peak O_2_ uptake). These latter findings suggest that abdominal binding influences the overall exercise response by an increase in O_2_ transport capacity.

To our knowledge, this is the first report of respiratory mechanics during wheelchair exercise in individuals with SCI and the first to assess the effect of abdominal binding on exercise responses in cervical SCI. A novel finding was the sudden and sustained rise in end-expiratory lung volume (i.e., dynamic hyperinflation), despite no evidence of expiratory flow limitation. This finding is consistent with our previous observation for cervical SCI during arm-crank ergometry (41), but in contrast to that reported for able-bodied subjects during lower-body exercise whereby end-expiratory lung volume only increases above relaxation volume when subjects approach their mechanical limits to generate expiratory flow (2). It is not entirely clear whether the rise in end-expiratory lung volume is a consequence of expiratory muscle weakness (40) or merely the “normal” response to upper-body exercise (11). The expiratory muscle paralysis that accompanies cervical SCI leads to an increased recruitment of nontypical accessory muscles of expiration (e.g., pectoralis major) to expire below functional residual capacity (13). However, many of these accessory muscles are also involved as prime movers during wheelchair propulsion (28, 46). It is perhaps, therefore, unsurprising that hyperinflation prevails from the onset of exercise. The increased elastic recoil characteristics of the lung and chest wall at high lung volumes may be a mechanism by which individuals with cervical SCI are able to “passively” increase expiratory flow during exercise. Although abdominal binding did not prevent dynamic hyperinflation, it did cause a parallel downward shift in operating lung volumes at rest and throughout exercise. An increase in elastic recoil pressure with binding might be expected to increase driving pressure for tidal expiratory flow (9), albeit at a lower operating lung volume. Importantly, the downward shift in operating lung volumes did not appear to impose mechanical ventilatory constraints (see Fig. 5). Indeed, ventilatory reserve as a fraction of capacity was similar in both conditions, presumably owing to the binding-induced increases in vital capacity and maximal midexpiratory flows.

We reason that abdominal binding improves the ventilatory response to exercise through several interrelated factors. First, the binding-induced increase in intra-abdominal pressure during inspiration would be expected to increase expansion of the lower rib cage (31, 44). This, in turn, may improve overall gas exchange consequent to an increase in the ventilation-perfusion ratio of lung units (55). Indeed, the ventilatory equivalent for CO_2_ at peak exercise was reduced with binding (Table 3). Moreover, the physiological dead space ventilation estimated using the alveolar ventilation equation and an assumed anatomic dead space of 150 ml was more than halved (3.6 bound vs. 7.5 l/min unbound). Although the presumed increase in lower rib cage expansion has been attributed to an increase in appositional forces (31, 44), more recent evidence suggests that binding may enable the diaphragm to operate on a more effective portion of its length-tension relationship and thereby exert greater insertional force (57). This increase in mechanical advantage might be expected to decrease the propensity for diaphragm fatigue. In the unbound condition, the product of P̄_di_/P_di,max_ and T_I_/T_TOT_ (TTI_di_) during the final stage of submaximal exercise (0.20) exceeded “critical” values that have been proposed to elicit diaphragm fatigue in healthy, nondisabled individuals (>0.15) (7) and individuals with cervical SCI (>0.10) (33). With binding, however, there was a reduction in TTI_di_ (0.16) consequent to a slight increase in the capacity of the diaphragm to generate inspiratory pressure (P_di,max_). Although we acknowledge that the critical TTI_di_ concept may not apply directly to the hyperpnea of exercise (41), other factors known to influence energy demands, namely respiratory frequency and velocity of diaphragm shortening (V_T_/T_I_), were unaffected by binding. Thus the potential benefits of binding may revolve around an increase in the capacity and/or efficiency of the inspiratory muscles, which, in turn, would be expected to improve the overall energetics of these muscles. Despite the aforementioned changes in respiratory mechanics, dyspnea intensity ratings were essentially the same at any given power output and ventilation in both conditions. This latter finding suggests that binding-induced alterations in respiratory mechanics do not contribute importantly to exertional dyspnea in highly fit individuals with cervical SCI.

The changes in respiratory mechanics with binding were accompanied by significant changes in O_2_ uptake (8–12%) and blood lactate concentration (−16–19%) at high relative power outputs. By using a similar exercise protocol and subject population, Leicht et al. (27) reported within-day coefficients of variation of <6% for peak O_2_ uptake and <14% for peak lactate concentration. Thus the relatively large changes noted in the current study were likely to be “true” differences. The findings are an extension of our recent field-based study in which the distance covered during a 4-min maximal push test was significantly increased with binding and the blood lactate response was significantly reduced (50). In the only other study to investigate the influence of abdominal binding in athletes with SCI, Kerk et al. (25) found no change in O_2_ uptake during submaximal or maximal wheelchair exercise. The discrepancy may be because Kerk et al. (25) set the degree of abdominal compression based on a change in abdominal girth, whereas we adjusted the binder so that end-expiratory gastric pressure reached a level known to optimize resting cardiopulmonary function (52). Furthermore, Kerk et al. (25) studied athletes with high-thoracic SCI (≥T_6_), who, because of partial or full descending sympathetic control of the myocardium and upper-body vasculature, would be less likely to exhibit cardiovascular limitation during exercise and therefore benefit from binding.

The reason for the binding-induced increase in O_2_ uptake is not entirely clear. Power outputs were matched and push rates were similar across conditions. Moreover, we have shown that propulsion kinematics are not significantly altered with binding (50). It seems unlikely, therefore, that the greater increase in O_2_ uptake could be accounted for by an increase in the amount of active musculature and/or a decrease in mechanical efficiency. A potential explanation relates to an increase in work (and O_2_ cost) of breathing, as suggested by the slightly elevated tidal swings in transdiaphragmatic pressure with binding. In healthy nondisabled subjects, who would be expected to achieve much higher levels of ventilation than individuals with cervical SCI, the O_2_ cost of breathing during maximal whole body exercise averages 8–10% of total O_2_ uptake (1). Thus, although an increase in respiratory muscle work might have accounted for a small proportion of the increase in total O_2_ uptake with binding, we doubt whether this could have contributed a significant amount to the 12% increase at peak exercise.

A more likely explanation for the binding-induced increase in O_2_ uptake relates to an improvement in central hemodynamics. Although our study was not specifically designed to address this issue, our observations do merit discussion. The increases in abdominal pressure due to application of the binder (Fig. 3) may be expected to decrease vascular compliance, increase mean vascular pressure, and therefore increase stroke volume. The increase in end-expiratory and end-inspiratory abdominal pressures might also be expected to increase the degree of driving pressure for venous return during tidal breathing. In this regard, Aliverti et al. (3, 4) showed that the circulatory function of the diaphragm in nondisabled subjects is greatly enhanced by the action of the abdominal muscles. Increases in abdominal pressure with quiet diaphragmatic breathing were shown to expel blood from the splanchnic vascular bed (3, 4). Moreover, increases in abdominal pressure resulting from expulsive maneuvers performed by simultaneous contractions of the diaphragm and abdominal muscles were shown to augment the circulatory function of the diaphragm (3, 4). These findings are relevant in so far as individuals with cervical SCI lack central sympathetic control (42). As a result, blood pooling occurs in nonactive vascular beds, including the splanchnic region (43). This, in turn, may limit O_2_ transport capacity by restricting the ability to increase venous return and stroke volume (22). In the current study, the increase in heart rate for a given increase in O_2_ uptake was reduced by ∼20% with binding (Fig. 2) and the O_2_ pulse at high relative exercise intensities was increased by ∼16%. These latter findings are consistent with our observation of an improvement in left-ventricular function at rest (52) and are highly suggestive of a binding-induced increase in stroke volume during exercise (56).

Another potential mechanism for the proposed increase in stroke volume with binding relates to the downward shift in operating lung volumes. In the unbound condition, end-inspiratory lung volume averaged 83% of total lung capacity, and three subjects achieved >90% (see Fig. 5). Conceivably, this severe level of dynamic hyperinflation may place a constraint on ventricular preload during inspiration by a compressive effect of the lung on the cardiac fossa and the inferior and superior vena cava (29, 39). In turn, the decrease in end-inspiratory lung volume with binding may have reduced mechanical compression of the heart and great vessels, thereby resulting in an elevation of cardiac filling and stroke volume. An effect of changing operating lung volumes on cardiac function might be particularly relevant for individuals with cervical SCI because lung compliance is reduced in this population (37). Thus binding may exert a cardiogenic benefit, both directly via an abdominothoracic translocation of blood and indirectly via an attenuation of dynamic hyperinflation. The consequent increase in blood flow to working muscles may explain the modest but consistent reductions in blood lactate concentration at high exercise intensities. This effect of increasing blood flow may be attributed to alterations in metabolism resulting from increases in O_2_ delivery and metabolite removal (6).

Despite a greater peak O_2_ uptake with binding, peak power output was similar across conditions. This appears to suggest that exercise tolerance was limited more by the ability of the muscles to use O_2_ (i.e., peripheral factors) than the capacity to transport O_2_ (i.e., central factors). Alternatively, the exercise protocol (i.e., rapid increases in gradient with a constant speed) may have been suboptimal for eliciting a true peak response, therefore masking our ability to detect a binding-induced increase in exercise tolerance. We recently showed that peak heart rate is significantly higher during a field-based endurance test compared with a laboratory-based incremental treadmill test (54). Moreover, when the subjects in the current study were tested using the field-based test, every subject demonstrated a binder-induced improvement in endurance performance (50). Further support for our postulate that rapid increases in gradient may not be suitable for detecting changes in exercise tolerance stems from the finding that elite hand-cyclists with cervical SCI perform worse against their counterparts with thoracic SCI during uphill pushing vs. on the flat (49).

In conclusion, abdominal binding shifts tidal breathing to lower lung volumes with no effect on flow limitation, symptom intensities, or exercise tolerance. Changes in respiratory mechanics with binding may raise muscle blood flow and O_2_ delivery during maximal exercise by an increase in cardiac filling and output. Potential mechanisms include a translocation of blood from the abdomen to the heart and a decrease in mechanical compression of the heart and great vessels via a shift of tidal breathing to lower lung volumes. The physiological relevance of the findings is that O_2_ transport capacity in cervical SCI may be limited by an inability of the cardiovascular system to further increase cardiac output. From a practical perspective, binder-induced improvements in central circulatory function may enable individuals with cervical SCI to achieve greater cardiovascular adaptations to exercise training. Future studies should include direct measurements of central and peripheral hemodynamics to fully characterize the acute and chronic effects of abdominal binding on O_2_ delivery and utilization during exercise.

## GRANTS

The study was funded by UK Sport through the Ideas4Innovation Programme.

## DISCLOSURES

No conflicts of interest, financial or otherwise, are declared by the author(s).

## AUTHOR CONTRIBUTIONS

Author contributions: C.R.W., V.L.G.-T., I.G.C., and L.M.R. conception and design of research; C.R.W. performed experiments; C.R.W. and L.M.R. analyzed data; C.R.W. and L.M.R. interpreted results of experiments; C.R.W. prepared figures; C.R.W. and L.M.R. drafted manuscript; C.R.W., V.L.G.-T., I.G.C., and L.M.R. edited and revised manuscript; C.R.W., V.L.G.-T., I.G.C., and L.M.R. approved final version of manuscript.
